# The Role of Novel Digital Clinical Tools in the Screening or Diagnosis of Obstructive Sleep Apnea: Systematic Review

**DOI:** 10.2196/47735

**Published:** 2023-07-26

**Authors:** Miguel Duarte, Pedro Pereira-Rodrigues, Daniela Ferreira-Santos

**Affiliations:** 1 Faculty of Medicine University of Porto Porto Portugal; 2 Department of Community Medicine, Information and Decision Sciences (MEDCIDS), Faculty of Medicine, University of Porto Porto Portugal; 3 Center for Health Technology and Services Research (CINTESIS), Faculty of Medicine, University of Porto Porto Portugal

**Keywords:** obstructive sleep apnea, diagnosis, digital tools, smartphone, wearables, sensor, polysomnography, systematic review, mobile phone

## Abstract

**Background:**

Digital clinical tools are a new technology that can be used in the screening or diagnosis of obstructive sleep apnea (OSA), notwithstanding the crucial role of polysomnography, the gold standard.

**Objective:**

This study aimed to identify, gather, and analyze the most accurate digital tools and smartphone-based health platforms used for OSA screening or diagnosis in the adult population.

**Methods:**

We performed a comprehensive literature search of PubMed, Scopus, and Web of Science databases for studies evaluating the validity of digital tools in OSA screening or diagnosis until November 2022. The risk of bias was assessed using the Joanna Briggs Institute critical appraisal tool for diagnostic test accuracy studies. The sensitivity, specificity, and area under the curve (AUC) were used as discrimination measures.

**Results:**

We retrieved 1714 articles, 41 (2.39%) of which were included in the study. From these 41 articles, we found 7 (17%) smartphone-based tools, 10 (24%) wearables, 11 (27%) bed or mattress sensors, 5 (12%) nasal airflow devices, and 8 (20%) other sensors that did not fit the previous categories. Only 8 (20%) of the 41 studies performed external validation of the developed tool. Of these, the highest reported values for AUC, sensitivity, and specificity were 0.99, 96%, and 92%, respectively, for a clinical cutoff of apnea-hypopnea index (AHI)≥30. These values correspond to a noncontact audio recorder that records sleep sounds, which are then analyzed by a deep learning technique that automatically detects sleep apnea events, calculates the AHI, and identifies OSA. Looking at the studies that only internally validated their models, the work that reported the highest accuracy measures showed AUC, sensitivity, and specificity values of 1.00, 100%, and 96%, respectively, for a clinical cutoff AHI≥30. It uses the *Sonomat*—a foam mattress that, aside from recording breath sounds, has pressure sensors that generate voltage when deformed, thus detecting respiratory movements, and uses it to classify OSA events.

**Conclusions:**

These clinical tools presented promising results with high discrimination measures (best results reached AUC>0.99). However, there is still a need for quality studies comparing the developed tools with the gold standard and validating them in external populations and other environments before they can be used in clinical settings.

**Trial Registration:**

PROSPERO CRD42023387748; https://www.crd.york.ac.uk/prospero/display_record.php?RecordID=387748

## Introduction

### Background

Obstructive sleep apnea (OSA) is a sleep-related breathing disorder characterized by transitory periods of breathing cessation due to partial (hypopnea) or complete (apnea) obstruction of the respiratory tract that affects ventilation during sleep. Repeated episodes of upper airway obstruction during sleep may, understandably, result in sleep fragmentation, nonrestorative sleep, and excessive daytime somnolence [1]. Furthermore, it has a great impact on multiple organ systems and is associated with hypertension, cardiovascular morbidities (eg, arrhythmias, coronary artery, and cerebrovascular diseases), and decrements in cognitive function [2].

The prevalence of this disease varies greatly depending on the population being studied and on how OSA is defined (eg, testing methodology, scoring criteria used, and apnea-hypopnea index [AHI] threshold) [3]. Despite the heterogeneity in population prevalence studies, this number is high, as nearly one-seventh or one billion of the world’s adult population is thought to have some degree of OSA [4-6]. These numbers have been on an upward trajectory, partially because of the increasing number of individuals with excess weight and obesity in high-income countries, as this is one of the causal factors of this pathology [6,7]. Nevertheless, despite being a highly prevalent disease, many cases remain undiagnosed and untreated, resulting in a decrease in quality of life, along with an increase in the incidence of adverse events and overall mortality [8].

The current gold-standard method for the diagnosis of OSA is overnight polysomnography, which takes place in a sleep laboratory with the attendance of a sleep technician [3], and where oxygen saturation, oronasal airflow, respiratory movement, electroencephalogram, body position, electromyogram, electrooculogram, and electrocardiogram are recorded [3,9].

Traditionally, sleep studies have been categorized into type I (or polysomnography), type II, type III, and type IV. Contrary to type I, types II to IV are unattended. Type II studies use the same number of monitoring sensors as the gold standard but are performed outside of the sleep laboratory, normally at the patient’s home. Unfortunately, they lack technical quality because problems such as sensor displacement or malfunction cannot be addressed by sleep technicians. Type III studies, also known as home sleep apnea tests (HSATs), have already been validated and use devices that measure otherwise limited cardiopulmonary parameters: 2 respiratory variables (eg, airflow and breathing effort), oxygen saturation, and a cardiac variable (eg, heart rate [HR] or electrocardiogram). Finally, type IV sleep studies are the most limited type of sleep study, using devices that measure only 1 or 2 parameters, typically HR or oxygen saturation [3].

Although the polysomnography provides detailed and highly accurate results, it is a time-consuming, labor-intensive, and expensive test [6], as it requires the patient to stay overnight in the sleep laboratory, a sleep technician to attend the study, and manual scoring of the data to produce the results, just to list some disadvantages [3]. This causes sleep laboratories to be unable to keep up with demand, often with long waiting lists and inaccessibility to a large part of the population [10].

The use of digital tools and innovative devices is a rapidly expanding area of research and has the potential to revolutionize the way health services are delivered, increasing access to health care in an easier way and at lower costs [11]. They can be an invaluable addition for health care professionals, as they provide many different functions, ranging from clinical decision support systems to data collection [12].

Wearable devices provide a level of unobtrusiveness that is not achievable with standard techniques, conceivably allowing faster OSA screening, along with improved long-term characterization and follow-up because of the possibility of day-to-day use. Subsequently, research on the use of these instruments in the diagnosis of OSA has been growing rapidly in recent years, with numerous vital signs and sleep parameters being monitored and strategies being used [13]. For example, some rely on movement analysis during sleep using accelerometers (actigraphy), snoring audio processing using tracheal and ambient microphones, and oxygen saturation measurement [14].

In contrast, smartphone-based health care platforms are emerging as an innovative solution owing to their ability to integrate, in the same device or in combination with other wireless wearable devices, several of the essential sensors to obtain the desired physiological variables for sleep-related disease diagnosis [14]. In addition, because of their ability to monitor sleep over long periods in the home setting, wrist-worn sleep devices, such as smartwatches and fitness trackers, are gaining attention from the sleep medicine community, using photoplethysmography, microphones, accelerometers, HR, or oximetry data [15]. Sheet-shaped or under-the-mattress sensors are also upcoming technologies that use pressure sensors for the detection of respiratory efforts based on accompanying thoracic movements [16], which spares the patient from being restrained by attached sensors and consequently allows a more natural and comfortable sleep experience.

Therefore, the integration of these contemporary and latest devices and platforms has the potential to improve patient care and grant better access to screening or diagnostic tests, allowing for quicker diagnosis, monitoring, and treatment of patients with OSA [13].

### Objective

Given the shortcomings of the current gold standard and the promising features of the new innovative digital clinical tools, this systematic review aimed to identify, gather, and analyze the most accurate digital tools used for OSA screening or diagnosis in the adult population. We intend to identify individuals with a higher risk of developing this disease, which would benefit the most from a full in-laboratory polysomnography to confirm the diagnosis, relieving some of the pressure on this field, following a rule-in approach.

## Methods

This systematic review was carried out according to the PRISMA (Preferred Reporting Items for Systematic Reviews and Meta-Analyses) guidelines [17], and the protocol was registered in the PROSPERO under the reference CRD42023387748.

### Search Strategy and Selection Criteria

A comprehensive literature search, without any restrictions, was conducted using the PubMed, Scopus, and Web of Science databases for articles published until November 2022. Specific queries were used for each platform, which can be found in Multimedia Appendix 1. Subsequently, a manual search was performed using relevant references from the included studies and relevant reviews on the matter. If there was no access to the full-text article on the web, the respective authors were contacted to obtain it.

Articles were independently selected by 2 reviewers (blinded to each other’s assessment, MD and DFS), applying predefined criteria to each article’s title and abstract, and in the second phase, to the integral texts of the selected articles. Divergent opinions were resolved by consensus. These processes were conducted using Rayyan (Qatar Computing Research Institute), a web and mobile app that helps expedite the initial screening of articles for systematic reviews [18].

Included in this review were studies that reported on adult patients with suspected OSA or OSA diagnosis (population) and assessed the accuracy of digital clinical tools for the screening or diagnosis of OSA (exposure and comparator) while having polysomnography as a gold standard (outcome). Studies that evaluated the accuracy of digital tools in pregnant women or the pediatric population that used HSAT or other types of sleep studies as the reference test, as well as interventions using only one portion of the data obtained by polysomnography as the index test (eg, pulse oximetry, electroencephalography, and electrocardiogram), were excluded.

### Data Extraction

Once the articles were selected, data were extracted to a prespecified Microsoft Excel spreadsheet by 2 reviewers (MD and DFS) blinded to each other’s assessment, which included the following: (1) article information: title, authors, publication date, country, and journal and (2) methods: participant selection, sample size, execution or nonexecution of in-laboratory polysomnography, prevalence of OSA, type of digital tool analyzed in comparison with polysomnography, inclusion and exclusion criteria, and potential bias.

To enhance the comprehension and analysis of the data from all included studies, we grouped the digital clinical tools into five categories: (1) smartphone-based tools, (2) wearable tools, (3) bed or mattress sensors, (4) nasal airflow devices, and (5) other digital tools. The last category was created to avoid further subdivisions, as some tools did not fit the previous ones.

For each type of tool, specific data were extracted, including population (n), clinical cutoff values for the diagnosis of OSA and severity classification (AHI), area under the curve (AUC), sensitivity, and specificity values.

Moreover, an additional division was made regarding the validation of the developed tool. If the studies merely developed a digital tool or developed and tested it on the same collected population, the results were presented for that group and represented by “D”—derivation group—in the tables. If the study was developed and validated on a different population, those results were presented and represented by “V”—validation group. In addition, a further subdivision could be performed regarding subject- or event-wise validation because the results obtained from each method should not be directly compared. Even so, given the few included studies that performed event-wise validation, this subdivision was not considered. However, these studies are mentioned with a footnote in the respective tables.

Studies were presented by the year of publication within each category. Any missing information is reported in the tables of the *Results* section by “—” (meaning “not available”), and the AHI cutoff for which the best metrics were obtained is marked in italics. A special note to one article presents the results for several sensor positions, where we only show the best results. In addition, each manuscript was checked for the definition of hypopnea, namely, the percentage of desaturation, but as this definition was not clear in most of the studies, we did not consider it further.

Finally, as we intended to identify and select patients with a high probability of having OSA suitable to perform polysomnography, tools with high specificity values were considered the best, following a rule-in approach.

### Risk of Bias

At 2 points in time, 2 reviewers (MD and DFS) assessed the risk of bias in all 41 included studies. It was performed by analyzing and answering a total of 10 questions from the Joanna Briggs Institute critical appraisal tool for diagnostic test accuracy studies [19]. All answers can be found in the tables and are represented by symbols according to their risk of bias. A green minus sign is presented in the table if a low risk of bias was found for a question. A red plus sign is presented if a substantial risk of bias is found. A yellow question mark is presented if the risk of bias was unclear. If the question did not apply to our specifically analyzed studies, they were indicated as *not applicable*.

Regarding the questions, each was replaced with the letter Q in the tables, followed by the number of the question: Q1, “Was a consecutive or random sample of patients enrolled?” Q2, “Was a case-control design avoided?” Q3, “Did the study avoid inappropriate exclusions?” Q4, “Were the index test results interpreted without knowledge of the results of the reference standard?” Q5, “If a threshold was used, was it prespecified?” Q6, “Is the reference standard likely to correctly classify the target condition?” Q7, “Were the reference standard results interpreted without knowledge of the results of the index test?” Q8, “Was there an appropriate interval between the index test and reference standard?” Q9, “Did all patients receive the same reference standard?” and Q10, “Were all patients included in the analysis?”

Given that the index test (digital tool) and the gold standard (polysomnography) were performed simultaneously in all studies included, Q8 was *not applicable* to any of the designs.

## Results

### Overview

**Figure 1:**
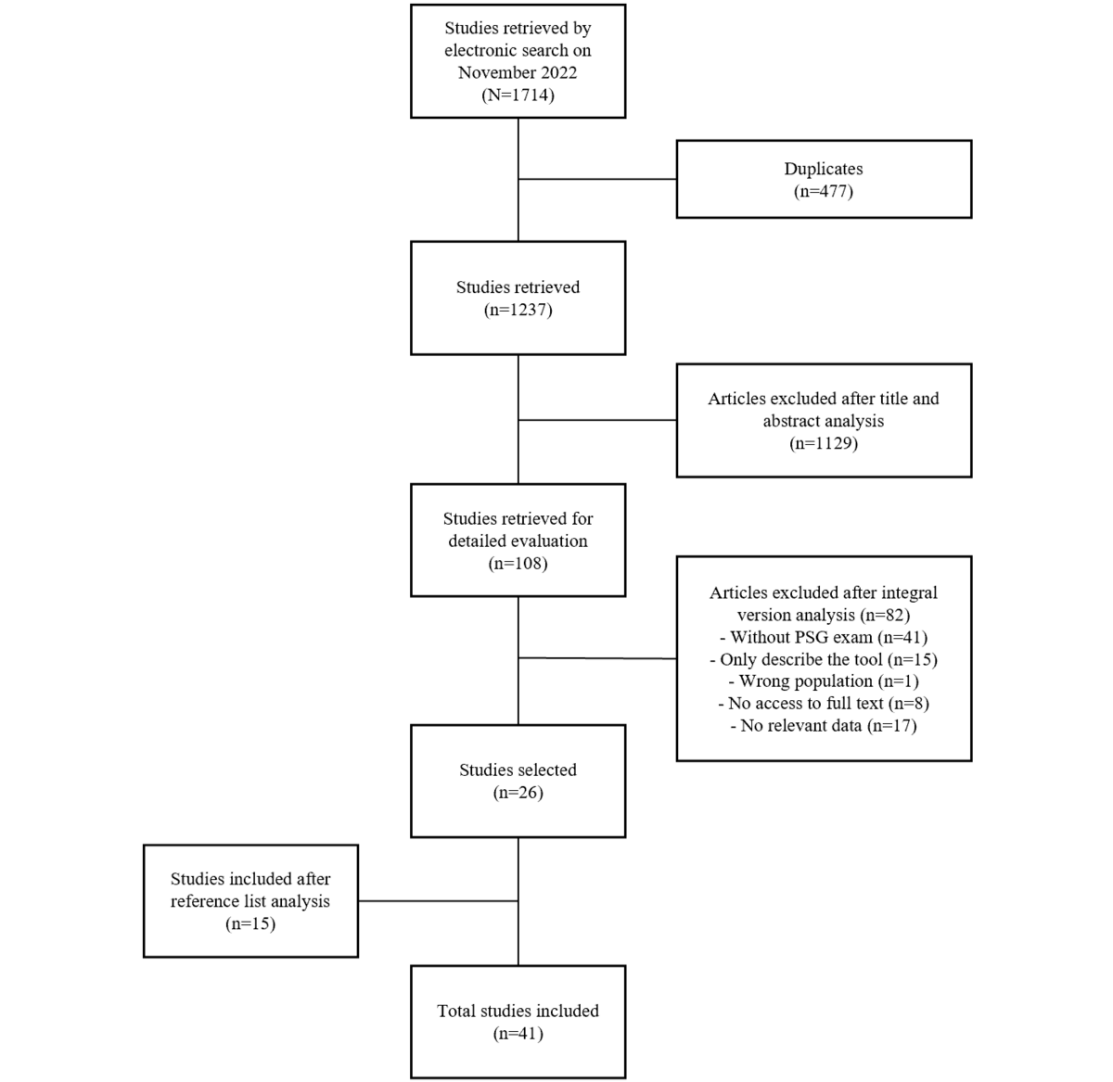
Flow diagram showcasing the article selection process. PSG: polysomnography.

We retrieved 1714 articles, of which 477 were duplicates. From the 1237 articles, after in-depth scrutiny, we retained 41 papers that met the inclusion criteria, as shown in Figure 1.

Disagreements were observed between the reviewers during both phases of the analysis. The overall rate of concordance in the title and abstract screening was 87%, whereas that in the integral version was 92%.

The gold-standard diagnostic test, polysomnography, was performed in all the studies included in our review. Some studies were unclear about the overall context in which the polysomnography was performed and did not report any details about the polysomnography data collection (eg, setting, equipment used, number of channels, and overall conditions); therefore, we excluded them from the analysis.

The oldest digital clinical tool was developed in 2002 and consists of a small, lightweight device worn underneath the nose and above the upper lip, which identifies and counts nasal airflow cessations through the night and uses it to predict the probability of OSA [20]. In contrast, in 2022, a variety of tools were developed and tested, namely a smartphone-based method [21], a wearable adhesive patch [22], 2 radar devices [23,24], and an audio recorder [25]. The frequency distribution of the tools included in our systematic review is shown in Table 1.

**Table 1:** Frequency distribution of the tools included in our systematic review (n=41).

Digital tools	Number of articles, n (%)
Smartwatch	4 (10)
Smart pillow	1 (2)
Smartphone	7 (17)
Ring	1 (2)
Respiration belt	1 (2)
Radar	5 (12)
Neck device	1 (2)
Nasal air flow	5 (12)
Microphones	2 (5)
Mattress sensor	10 (24)
Garment	1 (2)
Bone conducted vibration	1 (2)
Adhesive patch	2 (5)

Overall, the largest sample size was 620 patients [26], whereas the smallest consisted of only 5 patients [27], with a median sample size of 53 patients with suspected OSA. The overall prevalence of OSA was reported in 21 of the 41 included studies, with values ranging from 51% [28] to 100% [29].

AHI was used to define OSA presence or absence, as well as to stratify patients by severity level, with the chosen cutoff in each paper stated in the respective table. A considerable number of studies did not report the cutoff value (12/41, 29%), whereas among those who chose only 1 definition cutoff, the most frequent was AHI≥15 (8/41, 19%), followed by AHI≥5 (3/41, 7%). One study [30] used a different cutoff from the rest, AHI≥20, whereas another study [31] performed a comparison between the different severity-level subgroups using AHI=5, AHI=15, and AHI=30 instead of comparing the presence or absence of the disease for a specific AHI cutoff, thus impairing the comparison with the rest. The remaining studies (17/41, 41%) presented several severity cutoffs and corresponding results for each, often AHI≥5, AHI≥15, and AHI≥30.

### Smartphone-Based Tools

Of the 41 included articles, 7 (17%) analyzed the screening or diagnostic ability of smartphone-based tools (Table 2) by using 1 or more of the sensors incorporated in the smartphones. A total of 3 studies used a microphone to capture audio signals to detect the patient’s respiratory effort, which were then processed and analyzed to predict the diagnosis [21,32,33], with 1 study [33] concomitantly using the smartphone’s accelerometer. In contrast, 2 studies created apps as a form of screening using models that can predict the risk of OSA from patient variables [26] or process signals to detect patterns of sleep-disordered breathing [28]. The remaining 2 studies transformed the smartphone into a sonar device that emits sound and then captures its reflection, using it to classify the respiratory movements of the patient [34,35].

OSA definition was stated in only 43% (3/7) of studies, all of which used an AHI≥15 cutoff. The largest sample size recorded was 620 patients, whereas the smallest sample comprised only 15 patients. The prevalence of OSA was reported in 71% (5/7) of articles, ranging from 51% to 70%. Regarding the studies that only performed internal validation, the best AUC, sensitivity, and specificity values were 0.95, 94%, and 97%, respectively, for an AHI≥15 cutoff [28]. As for the studies that performed external validation, 2 studies were found, namely, Narayan et al [32] that reported AUC, sensitivity, and specificity values of 0.87, 94%, and 63%, respectively, and Tiron et al [35] that reported values of 0.92 (95% CI 0.85-0.95), 88% (95% CI 67%-95%), and 80% (95% CI 68%-89%), respectively.

Table 3 shows the risk of bias for studies within this category. In the patient selection domain, 1 study [33] had a high risk of bias, 3 studies had a low risk of bias [21,28,34], and 3 others had an unclear risk of bias [26,32,35]. For the index test domain, 4 studies presented a high risk [21,26,33,34], namely in questions Q4 and Q5, with 2 studies having a low risk [28,32], and 1 study being unclear [35].

**Table 2:** Study characteristics of the smartphone-based tools category^a^.

Study, year	Digital tool	OSA^b^ definition	Sample size, n (group type: D=derivation group, V=validation group)	OSA prevalence, n (%)	Area under the curve, (95% CI)	Sensitivity, % (95% CI)	Specificity, % (95% CI)
Al-Mardini et al [33], 2014	Smartphone (audio; accelerometer)	—^c^	15 (D)	8 (53)	—	100 (—)	86 (—)
Nandakumar et al [34], 2014	Smartphone (sonar; audio)	—	37 (D)	26 (70)	—	—	—
Narayan et al [32], 2018	Smartphone (audio)	AHI^d^≥15	32 (D); 59 (V)	48 (53)	0.87 (—)	94 (—)	63 (—)
Lyon et al [28], 2019	Smartphone (app; sonar)	AHI≥15	162 (D)	83 (51)	0.95 (—)	94 (—)	97 (—)
Haberfeld et al [26], 2020	Smartphone (app)	—	620 (D)	357 (58)	Male: 0.61 (—); female: 0.62 (—)	Male: 86 (—); female: 56 (—)	—
Tiron et al [35], 2020	Smartphone (sonar; audio)	AHI≥15	128 (D); 120 (V)	—	0.92 (0.85-0.95)	88 (77-95)	80 (68-89)
Castillo-Escario et al [21], 2022^e^	Smartphone (audio)	—	16 (D)	—	0.88 (—)	72 (—)	89 (—)

^a^When external validation was performed, the results are only presented for the validation group.

^b^OSA: obstructive sleep apnea.

^c^Not available.

^d^AHI: apnea-hypopnea index.

^e^Used event-wise validation.

**Table 3:** Risk of bias for smartphone-based tools category using the Joanna Briggs Institute critical appraisal checklist for diagnostic test accuracy studies.

Study, year	Risk of bias									
	Patient selection			Index test						
	Q1	Q2	Q3	Q4	Q5	Q6	Q7	Q8	Q9	Q10
Al-Mardini et al [33], 2014	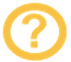 ^a^	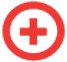 ^b^	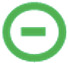 ^c^	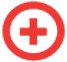	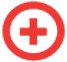	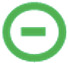	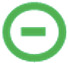	N/A^d^	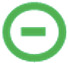	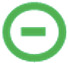
Nandakumar et al [34], 2014	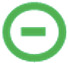	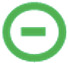	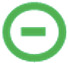	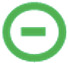	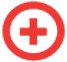	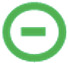	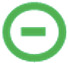	N/A	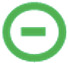	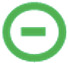
Narayan et al [32], 2018	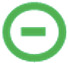	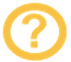	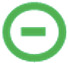	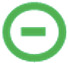	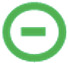	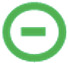	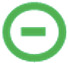	N/A	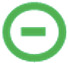	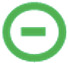
Lyon et al [28], 2019	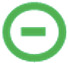	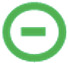	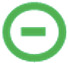	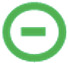	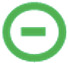	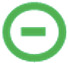	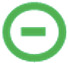	N/A	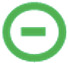	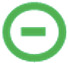
Haberfeld et al [26], 2020	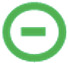	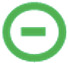	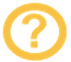	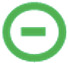	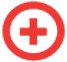	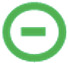	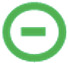	N/A	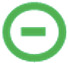	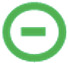
Tiron et al [35], 2020	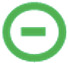	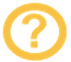	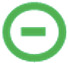	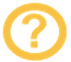	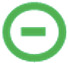	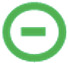	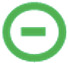	N/A	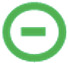	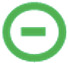
Castillo-Escario et al [21], 2022	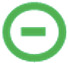	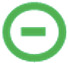	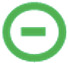	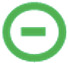	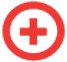	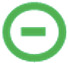	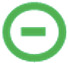	N/A	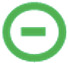	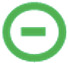

^a^Indicates unclear risk of bias.

^b^Indicates high risk of bias.

^c^Indicates low risk of bias.

^d^N/A: not applicable.

### Wearable Tools

Table 4 shows the 10 (24%) of the 41 wearable tools included in our systematic review. The device used in 4 of them was a smartwatch, making use of their built-in sensors, such as photoplethysmography signals or an accelerometer [36-39]. For the other wearables, there was a garment or body-worn sensor that recorded electrocardiogram, thoracic movements, and positional signals [40]; a respiration belt that registered thoracic movement during respiration [41]; and a neck device that recorded snoring and position using a microphone and accelerometer, respectively [42]. In addition, 2 adhesive patches were included: one that recorded electrocardiogram and actigraphy signals that were patched to the chest [43], and one that recorded blood saturation on the hand using a photoelectric reflex sensor [22]. Finally, a ring device capturing oxygen saturation, photoplethysmography signals, and accelerometer data are also shown in this table [44].

OSA definition was stated in 70% (7/10) of studies. The AHI≥15 cutoff was used in 3 of them, whereas the rest presented 2 or 3 severity cutoffs (AHI≥5, AHI≥15, and AHI≥30). The largest sample size was 404 patients, whereas the smallest had only 20 patients. The prevalence of OSA was reported in 50% (5/10) of articles and varied between 54% and 85%. Considering studies that only performed internal validation, the best AUC, sensitivity, and specificity values were 0.93, 86% (95% CI 57%-98%), and 100% (95% CI 54%-100%), respectively, for an AHI≥15 cutoff [38]. Once again, 2 studies [36,42] also performed external validation in a distinct group. Levendowski et al [42] reported sensitivity and specificity values of 78% and 92%, respectively, for AHI≥5 cutoff. In the study by Fedorin and Slyusarenko [36], these measures have not been reported. As previously stated, in 1 study, the authors presented results for 3 positions of the sensor, but we chose to include only the best position [43].

In Table 5, we can see that, for the patient selection domain, only 1 study showed a high risk of bias [43], with 6 studies having a low risk [22,36,37,40,41,44], and 3 studies being unclear [38,39,42]. As for the index test domain, 6 studies presented a high risk [36-38,40-42] among questions Q5 and Q10, and the other 4 studies showed a low risk of bias [22,39,43,44].

**Table 4:** Study characteristics of the wearable tools category^a^.

Study, year	Digital tool	OSA^b^ definition	Sample size, n (group type: D=derivation group, V=validation group)	OSA prevalence, n (%)	Area under the curve (95% CI)	Sensitivity, % (95% CI)	Specificity, % (95% CI)
Levendowski et al [42], 2014	Neck device	*AHI*^c^*>5*^d^; AHI≥10; AHI≥15	20 (D); 24 (V)	—^e^	—	*78 (—)*^d^; 81 (—); 87 (—)	*92 (—)*^d^; 87 (—); 88 (—)
Selvaraj and Narasimhan [43], 2014	Adhesive patch sensors (chest)	AHI≥15	53 (D)	32 (60)	—	94 (83-98)	79 (65-88)
Ben Azouz et al [40], 2018	Garment or body-worn sensor	—	32 (D)	—	—	—	—
Hayano et al [39], 2020	Smartwatch	AHI>15	41 (D)	22 (54)	—	82 (—)	89 (—)
Chen et al [37], 2021	Smartwatch	—	20 (D)	—	—	96 (—)	—
Ganglberger et al [41], 2021	Respiration belt	—	404 (D)	248 (61)	0.97 (—)	—	—
Chen et al [38], 2021	Smartwatch	AHI≥5; *AHI≥15*^d^; AHI≥30	20 (D)	17 (85)	0.81 (—); *0.93* (—)^d^; 0.80 (—)	77 (50-93); *86 (57-98)*^d^; 80 (44-98)	100 (29-100); *100 (54-100)*^d^; 80 (44-98)
Yeh et al [44], 2021	Ring	AHI≥5; AHI≥15; *AHI≥30*^d^	78 (D)	43 (55)	0.93 (0.88-0.98); 0.96 (0.90-1.00); *0.96 (0.92-1.00)*^d^	100 (92-100); 93 (77-99); *71 (42-92)*^d^	3 (0-15); 74 (59-85); *95 (87-99)*^d^
Fedorin and Slyusarenko [36], 2021	Smartwatch	AHI>15	107 (D); 69 (V)	—	—	—	—
Xu et al [22], 2022	Adhesive patch sensor (palm)	AHI≥5; *AHI≥15*^d^	196 (D)	—	0.95 (0.91-0.98); *0.95 (0.92-0.98)*^d^	93 (—); *92 (*—*)*^d^	77 (—); *89 (*—*)*^d^

^a^If the study used different clinical cutoff values for obstructive sleep apnea diagnosis, the results are only presented for the best-achieved cutoff and marked in italics. When external validation was performed, the results are only presented for the validation group.

^b^OSA: obstructive sleep apnea.

^c^AHI: apnea-hypopnea index.

^d^Best-achieved cutoff.

^e^Not available.

**Table 5:** Risk of bias for wearable tools category using Joanne Briggs Institute critical appraisal checklist for diagnostic test accuracy studies.

Study, year	Risk of bias										
	Patient selection				Index test						
	Q1	Q2	Q3	Q4		Q5	Q6	Q7	Q8	Q9	Q10
Levendowski et al [42], 2014	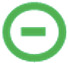 ^a^	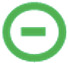	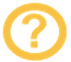 ^b^	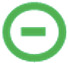		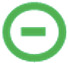	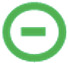	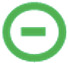	N/A^c^	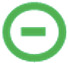	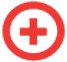 ^d^
Selvaraj and Narasimhan [43], 2014	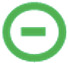	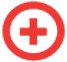	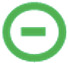	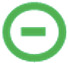		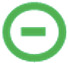	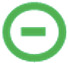	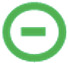	N/A	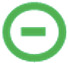	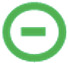
Ben Azouz et al [40], 2018	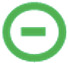	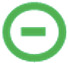	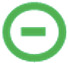	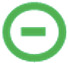		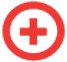	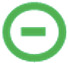	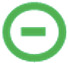	N/A	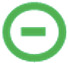	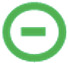
Hayano et al [39], 2020	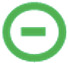	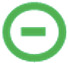	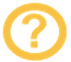	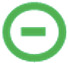		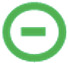	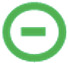	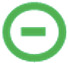	N/A	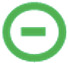	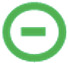
Chen et al [37], 2021	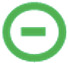	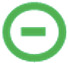	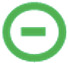	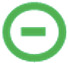		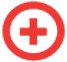	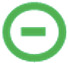	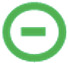	N/A	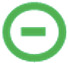	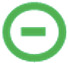
Ganglberger et al [41], 2021	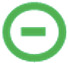	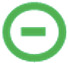	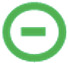	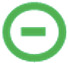		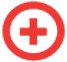	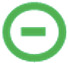	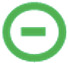	N/A	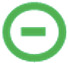	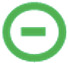
Chen et al [38], 2021	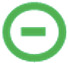	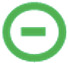	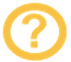	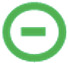		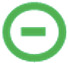	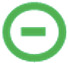	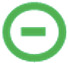	N/A	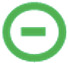	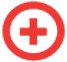
Yeh et al [44], 2021	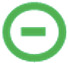	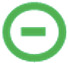	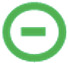	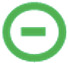		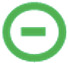	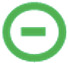	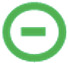	N/A	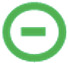	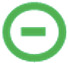
Fedorin and Slyusarenko [36], 2021	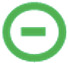	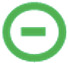	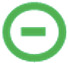	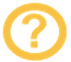		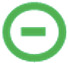	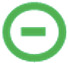	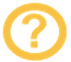	N/A	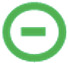	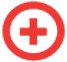
Xu et al [22], 2022	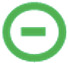	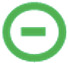	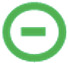	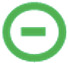		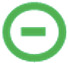	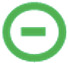	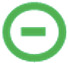	N/A	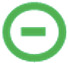	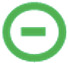

^a^Indicates low risk of bias.

^b^Indicates unclear risk of bias.

^c^N/A: not applicable.

^d^Indicates high risk of bias.

### Bed or Mattress Sensors

Bed or mattress-based sensors, for which the description can be found in Table 6, are also a big part of our pool of articles, with 11 (27%) of the 41 studies analyzing the performance and screening capability of these pressure-based sensing devices in the context of OSA [16,30,45-52]. In addition to under-the-mattress sensors, there is also a smart pillow paired with an oximeter, which offers diagnostic capability by detecting apnea events and interventional intent by being able to inflate and change its conformation and deobstruct the patient’s airway [29].

OSA definition was stated in 82% (9/11) of the studies. The majority presented 3 severity cutoffs (AHI≥5, AHI≥15, and AHI≥30), with 1 study using values of AHI>5, AHI>15, and AHI>20. For the rest, 2 studies used a cutoff of AHI≥5, 1 study used AHI≥15, and the other used AHI≥20. The largest sample size recorded was 366 patients, whereas the smallest sample size consisted of only 10. The prevalence of OSA was reported in 55% (6/11) of articles, ranging from 54% to 100%. With respect to studies that only performed internal validation, the best AUC, sensitivity, and specificity values were 1.00, 100%, and 96%, respectively, for an AHI≥30 cutoff [50]. This study used the *Sonomat*, a foam mattress that, aside from recording breath sounds, has pressure sensors that generate voltage when deformed, thus detecting respiratory movements, and using it to classify OSA events. In this category, only 1 study externally validated the device; Agatsuma et al [16] reported sensitivity and specificity values of 92% and 98%, respectively, for an AHI≥30 cutoff, with no mention of the AUC value.

Table 7 shows, in the patient selection domain, 3 studies with a high risk of bias [29,47,50], 6 with a low risk [16,30,45,48,49,51], and 2 with unclear risk [46,52]. In turn, in the index test domain, 4 studies had a high risk [29,47,51,52], namely in questions Q5 and Q10, and the other 7 studies presented a low risk of bias [16,30,45,46,48-50].

**Table 6:** Study characteristics of the bed or mattress sensors category^a^.

Study, year	Digital tool	OSA^b^ definition	Sample size, n (group type: D=derivation group, V=validation group)	OSA prevalence, n (%)	Area under the curve, (95% CI)	Sensitivity, % (95% CI)	Specificity, % (95% CI)
Agatsuma et al [16], 2009	Under-the-mattress sensor	AHI^c^≥5; AHI≥15; *AHI≥30*^d^	201 (D); 165 (V)	—^e^	—	100 (—); 100 (—); *92 (*—*)*^d^	41 (—); 90 (—); *98* (—)^d^
Tenhunen et al [52], 2013	Under-the-mattress sensor	AHI>5; *AHI>15*^d^; AHI>30	157 (D)	—	—	77 (—); *95 (*—*)*^d^; 94 (—)	81 (—); *92* (—)^d^; 82 (—)
Zhang et al [29], 2013^f^	Smart pillow + oximeter	—	40 (D)	40 (100)	—	—	—
Tsukahara et al [30], 2014	Under-the-mattress sensor	AHI≥20	101 (D)	—	—	90 (—)	90 (—)
Hwang et al [46], 2014	Under-the-mattress sensor	AHI >5; *AHI>15*^d^; AHI>20	32 (D)	26 (81)	0.98 (—); *0.99* (—)^d^; 0.98 (—)	100 (—); *100* (—)^d^; 92 (—)	75 (—); *92* (—)^d^; 92 (—)
Norman et al [50], 2014	Under-the-mattress sensor	AHI≥5; AHI≥15; *AHI≥30*^d^	43 (D)	35 (81)	0.94 (—); 0.97 (—); *1.00* (—)^d^	94 (—); 88 (—); *100* (—)^d^	77 (—); 91 (—); *96* (—)^d^
Mora et al [48], 2015	Under-the-mattress sensor	AHI≥5	24 (D)	13 (54)	—	—	—
Meng et al [47], 2016	Under-the-mattress sensor	*AHI≥5*^d^; AHI≥15; AHI≥30	131 (D)	—	*0.98* (—)^d^; 0.98 (—); 0.98 (—)	*95* (—)^c^; 90 (—); 90 (—)	*100* (—)^d^; 97 (—); 95 (—)
Davidovich et al [45], 2016	Under-the-mattress sensor	AHI≥15	96 (D)	64 (67)	—	88 (—)	89 (—)
Mosquera-Lopez et al [49], 2019	Under-the-mattress sensor	AHI>5	14 (D)	8 (57)	—	89 (—)	77 (—)
Sadek et al [51], 2020^f^	Under-the-mattress sensor	—	10 (D)	—	—	57 (—)	45 (—)

^a^If the study used different clinical cutoff values for the diagnosis of obstructive sleep apnea, the results are only presented for the best-achieved cutoff and marked in italics. When external validation was performed, the results are only presented for the validation group. Studies that used event-wise validation are mentioned in a footnote.

^b^OSA: obstructive sleep apnea.

^c^AHI: apnea-hypopnea index.

^d^Best-achieved cutoff.

^e^Not available.

^f^Used event-wise validation.

**Table 7:** Risk of bias for bed or mattress sensors category using the Joanna Briggs Institute critical appraisal checklist for diagnostic test accuracy studies.

Study, year	Risk of bias										
	Patient selection				Index test						
	Q1	Q2	Q3	Q4		Q5	Q6	Q7	Q8	Q9	Q10
Agatsuma et al [16], 2009	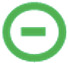 ^a^	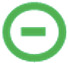	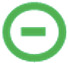	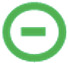		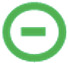	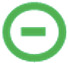	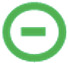	N/A^b^	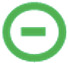	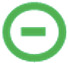
Tenhunen et al [52], 2013	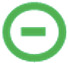	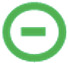	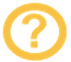 ^c^	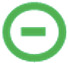		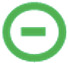	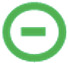	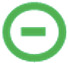	N/A	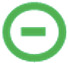	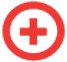 ^d^
Zhang et al [29], 2013	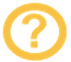	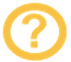	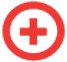	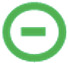		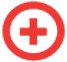	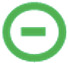	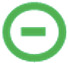	N/A	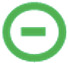	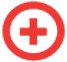
Tsukahara et al [30], 2014	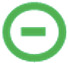	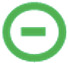	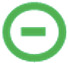	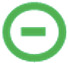		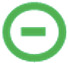	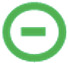	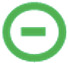	N/A	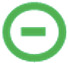	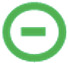
Hwang et al [46], 2014	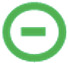	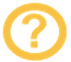	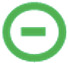	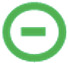		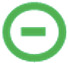	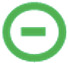	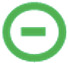	N/A	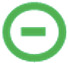	
Norman et al [50], 2014	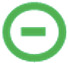	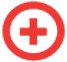	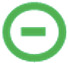	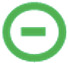		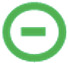	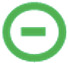	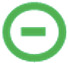	N/A	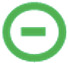	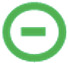
Mora et al [48], 2015	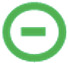	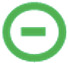	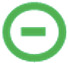	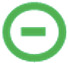		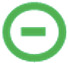	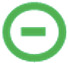	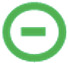	N/A	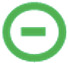	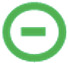
Meng et al [47], 2016	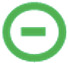	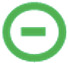	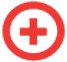	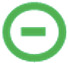		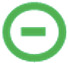	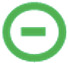	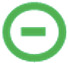	N/A	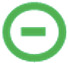	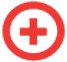
Davidovich et al [45], 2016	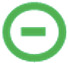	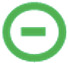	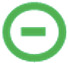	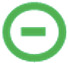		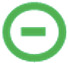	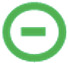	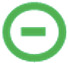	N/A	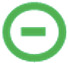	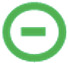
Mosquera-Lopez et al [49], 2019	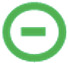	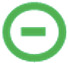	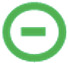	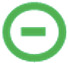		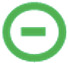	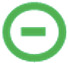	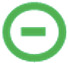	N/A	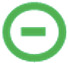	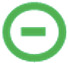
Sadek et al [51], 2020	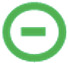	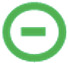	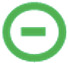	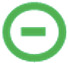		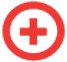	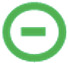	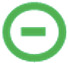	N/A	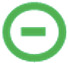	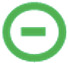

^a^Indicates low risk of bias.

^b^N/A: not applicable.

^c^Indicates unclear risk of bias.

^d^Indicates high risk of bias.

### Nasal Airflow Devices

In Table 8, we list the 5 (12%) nasal airflow devices out of the 41 tools. Of all the studies, 3 tested the accuracy of an under-the-nose pressure sensor named *SleepStrip* for detecting sleep events and diagnosing OSA [20,53,54]. Another study analyzed an under-the-nose pressure sensor that has not yet been marketed [27]. Finally, one piece of equipment was used to measure the nasal airflow using a nasal cannula [55].

OSA definition was only stated in the 3 studies using *SleepStrip*, with 1 of the studies using the usual cutoffs of AHI≥5, AHI≥15, and AHI≥30, and the other 2 studies using different cutoffs (AHI>10, AHI>20, and AHI>40). The largest sample size recorded was 288 patients, whereas the smallest sample comprised only 5. Only 1 (20%) out of the 5 studies reported values for the prevalence of OSA, registering 81% [53]. Contrary to the previous categories, none of the included studies performed external validations. The best reported AUC, sensitivity, and specificity values were 0.94 (95% CI 0.85-0.98), 94%, and 94%, respectively, for a clinical cutoff of AHI>40 [54].

This category showed the highest risk of bias (Table 9). All studies showed a high [54] or unclear [20,27,53,55] risk of bias in the patient selection domain. In the index test domain, there was only 1 study [53] with a low risk, whereas 4 studies [20,27,54,55] had a high risk of bias spanning questions Q5 and Q10.

**Table 8:** Study characteristics of nasal airflow devices category^a^.

Study, year	Digital tool	OSA^b^ definition	Sample size, n (group type: D=derivation group, V=validation group)	OSA prevalence, n (%)	Area under the curve, (95% CI)	Sensitivity, % (95% CI)	Specificity, % (95% CI)
Shochat et al [20], 2002	Under-the-nose pressure sensor *SleepStrip*	AHI^c^>10; AHI>20; *AHI>40*^d^	288 (D)	—^e^	—	86 (—); 80 (—); *80* (—)^e^	57 (—); 70 (—); *86* (—)^d^
Wong et al [55], 2008	Nasal cannula	—	34 (D)	—	—	—	—
Ozmen et al [54], 2011	Under-the-nose pressure sensor *SleepStrip*	AHI>10; AHI>20; *AHI>40*^d^	64 (D)		0.80 (0.68-0.89); 0.84 (0.72-0.92); *0.94* (*0.85-0.98*)^d^	83 (—); 80 (—); *94* (—)^d^	77 (—); 87 (—); *94* (—)^d^
Dinç et al [53], 2014	Under-the-nose pressure sensor *SleepStrip*	AHI≥5; AHI≥15; *AHI>30*^d^	41 (D)	33 (81)	0.77 (0.61-0.94); 0.82 (0.73-1.00); *0.91 (0.79-1.00)*^d^	54 (—); 44 (—); *45* (—)^d^	100 (—); 100 (—); *100* (—)^d^
Jin and Sánchez-Sinencio [27], 2015	Under-the-nose pressure sensor	—	5 (D)	—	—	—	—

^a^If the study used different clinical cutoff values for the diagnosis of obstructive sleep apnea, the results are only presented for the best-achieved cutoff and marked in italics. When external validation was performed, the results are only presented for the validation group.

^b^OSA: obstructive sleep apnea.

^c^AHI: apnea-hypopnea index.

^d^Best-achieved cutoff.

^e^Not available.

**Table 9:** Risk of bias for nasal airflow devices category using the Joanna Briggs Institute critical appraisal checklist for diagnostic test accuracy studies.

Study, year	Risk of bias										
	Patient selection				Index test						
	Q1	Q2	Q3	Q4		Q5	Q6	Q7	Q8	Q9	Q10
Shochat et al [20], 2002	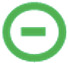 ^a^	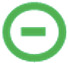	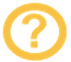 ^b^	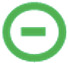		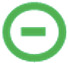	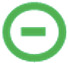	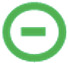	N/A^c^	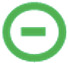	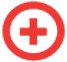 ^d^
Wong et al [55], 2008	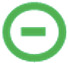	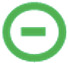	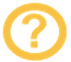	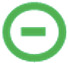		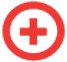	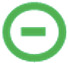	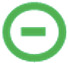	N/A	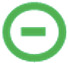	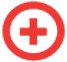
Ozmen et al [54], 2011	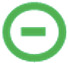	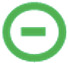	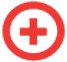	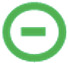		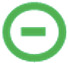	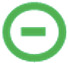	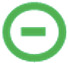	N/A	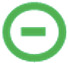	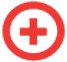
Dinç et al [53], 2014	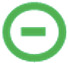	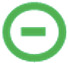	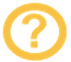	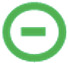		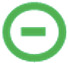	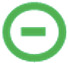	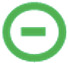	N/A	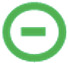	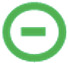
Jin and Sánchez-Sinencio [27], 2015	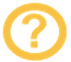	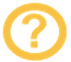	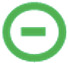	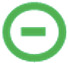		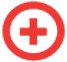	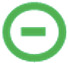	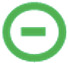	N/A	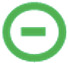	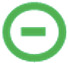

^a^Indicates low risk of bias.

^b^Indicates unclear risk of bias.

^c^N/A: not applicable.

^d^Indicates high risk of bias.

### Other Digital Tools

A total of 8 (20%) out of the 41 studies included tools that did not fit the previous categories, which are shown in Table 10. Among these studies, there are 5 that used radar technology to screen for OSA, which are novel devices in the shape of noncontact bedside sensors that use radio waves to detect and measure thoracic movement and respiration [23,24,56-58]. Audio recording using noncontact microphones was also featured in 2 studies, with posterior sleep sound analysis using algorithms and deep learning methods [25,59]. In addition, 1 study detected snoring using an unconventional method by capturing its vibration using a bone-conducted transducer [31].

OSA definition was stated in 88% (7/8) of the studies. Some of them used severity cutoffs to define OSA, with 4 studies using the usual 3 cutoff points (AHI≥5, AHI≥15, and AHI≥30), and 1 study using only 2 cutoffs (AHI≥10 and AHI≥15). A total of 1 study used AHI≥5 as the OSA definition and another study used AHI≥15. The largest sample size recorded was 359 patients, whereas the smallest had only 12 patients. The prevalence of OSA was reported in 50% (4/8) of the articles and varied between 79% and 85%. With regard to the studies that only performed internal validation, the best AUC, sensitivity, and specificity values were 0.97, 89% (95% CI 81%-93%), and 94% (95% CI 90%-97%), respectively, for a cutoff of AHI≥30 [58]. External validation was performed in 3 studies within this category. One of them applied the tool to a cohort of male participants aged between 18 and 70 years, with a clinical diagnosis of hypertension and receiving antihypertensive medication [56]. The other 2 studies [24,25] validated the tools in a different group of 59 and 2 patients with similar characteristics as the derivation group. Crinion et al [56] reported AUC, sensitivity, and specificity values of 0.85, 88%, and 67%, respectively, for an AHI≥15 cutoff, whereas the study by Wang et al [25] presented values of 0.99, 96%, and 92%, respectively, for an AHI≥30. By contrast, the study by Zhuang et al [24] did not report any discrimination measures.

The studies included in this category had a lower risk of bias in the entire assembly, as shown in Table 11. In the patient selection domain, the risk of bias was negligible, whereas in the index test domain, only 2 studies faltered in questions Q5 [24] and Q10 [59].

**Table 10:** Study characteristics of other digital tools category^a^.

Study, year	Digital tool	OSA^b^ definition	Sample size, n (group type: D=derivation group, V=validation group, T=test group, V=validation group)	OSA prevalence, n (%)	Area under the curve, (95% CI)	Sensitivity, % (95% CI)	Specificity, % (95% CI)
Alshaer et al [59], 2013	Face frame with a microphone attached	AHI^c^≥10; *AHI≥15*^d^	32 (D)	—^e^	—	100 (—); *89* (—)^d^	85 (—); *96* (—)^d^
Zaffaroni et al [57], 2013	Radar	AHI≥5; *AHI≥15*^d^; AHI≥30	74 (D)	60 (81)	0.90 (—); *0.97* (—)^d^; 0.96 (—)	98 (—); *90* (—)^d^; 84 (—)	47 (—); *92* (—)^d^; 89 (—)
Crinion et al [56], 2020	Radar	AHI≥15	67 (D); 55 (V)	53 (79)	0.85 (—)	88 (—)	67 (—)
Xin et al [31], 2021	Bone-conducted transducer	AHI=5; *AHI=15*^d^; AHI=30	28 (D)	23 (82)	0.91 (—); *1.00* (—)^d^; 1.00 (—)	91 (—); *100* (—)^d^; 92 (—)	100 (—); *100* (—)^d^; 100 (—)
Zhao et al [58], 2021	Radar—*OrbSense*	AHI≥5; AHI≥15; *AHI≥30*^d^	359 (D)	—	0.90 (—); 0.94 (—); *0.97* (—)^d^	96 (93-98); 90 (84-93); *89 (81-93)*^d^	56 (44-68); 81 (74-87); *94 (90-97)*^d^
Wei et al [23], 2022	Radar; ring	AHI≥5	67 (D)	57 (85)	0.85 (—)	100 (—)	70 (—)
Zhuang et al [24], 2022^f^	Radar	—	10 (D); 2 (V)	—	—	—	—
Wang et al [25], 2022	Audio recorder	AHI≥5; AHI≥15; *AHI≥30*^d^	116 (D); 19 (T); 59 (V)	—	0.94 (—); 0.98 (—); *0.99* (—)^d^	94 (—); 89 (—); *96* (—)^d^	83 (—); 96 (—); *92* (—)^d^

^a^If the study used different clinical cutoff values for the diagnosis of obstructive sleep apnea, the results are only presented for the best-achieved cutoff and marked in italics. When external validation was performed, the results are only presented for the validation group. Studies that used event-wise validation are mentioned in a footnote.

^b^OSA: obstructive sleep apnea.

^c^AHI: apnea-hypopnea index.

^d^Best-achieved cutoff.

^e^Not available.

^f^Used event-wise validation.

**Table 11:** Risk of bias for other digital tools category using the Joanna Briggs Institute critical appraisal checklist for diagnostic test accuracy studies.

Study, year	Risk of bias									
	Patient selection			Index test						
	Q1	Q2	Q3	Q4	Q5	Q6	Q7	Q8	Q9	Q10
Alshaer et al [59], 2013	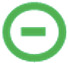 ^a^	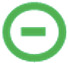	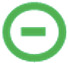	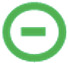	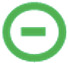	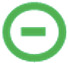	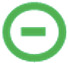	N/A^b^	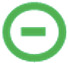	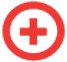 ^c^
Zaffaroni et al [57], 2013	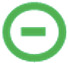	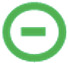	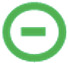	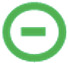	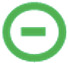	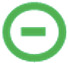	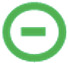	N/A	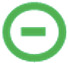	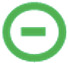
Crinion et al [56], 2020	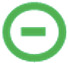	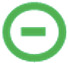	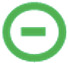	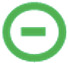	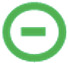	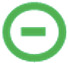	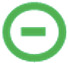	N/A	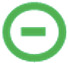	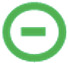
Xin et al [31], 2021	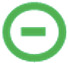	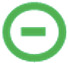	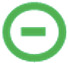	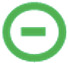	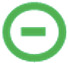	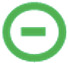	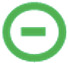	N/A	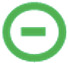	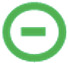
Zhao et al [58], 2021	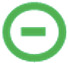	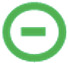	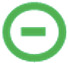	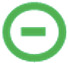	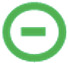	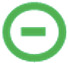	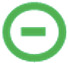	N/A	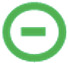	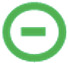
Wei et al [23], 2022	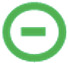	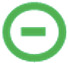	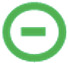	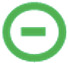	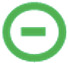	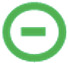	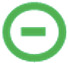	N/A	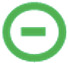	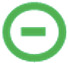
Zhuang et al [24], 2022	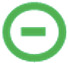	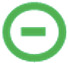	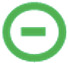	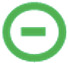	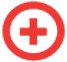	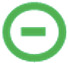	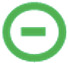	N/A	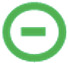	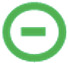
Wang et al [25], 2022	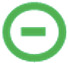	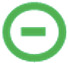	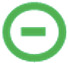	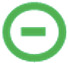	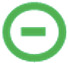	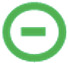	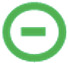	N/A	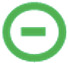	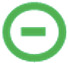

^a^Indicates low risk of bias.

^b^N/A: not applicable.

^c^Indicates high risk of bias.

## Discussion

### Principal Findings

As previously stated, this review aimed to gather the available evidence on upcoming digital tools in the screening or diagnosis of OSA, with a total of 41 tools that presented promising results, showing high discrimination measures (best results reaching AUC values higher than 0.99). This was done by grouping digital tools based on the technologies used, making discrimination measures comparable. Furthermore, we did not intend to replace the current gold standard, polysomnography, as the American Academy of Sleep Medicine guideline recommendations explicitly state that *“*clinical tools...should not be used to diagnose OSA in adults in the absence of polysomnography or home sleep apnea testing*”* [3]. However, digital devices and other mobile health tools can play an important adjuvant role in this process, which is also recognized by the American Academy of Sleep Medicine guidelines. It states that in *“*non-sleep clinic settings, these tools may be more helpful to identify patients who are at increased risk for OSA*”* [3] proving our rule-in approach. Recent reviews have assessed the potential use of digital tools in sleep-breathing disorders. The study by Behar et al [60] reviewed existing smartphone apps being used, particularly in OSA screening. They focused essentially on studies that applied questionnaires via an app or that used built-in smartphone sensors and characteristics, such as the accelerometer and the ability to record sleep sounds. However, it lacks a comparison of these proposed smartphone-based tools with the gold standard and the respective discrimination measures. The study by Kim et al [61] concentrated on the reliability of smartphones in the screening of moderate to severe OSA. In addition to the fact that our review covers a more versatile set of digital clinical tools, we also considered all diagnostic cutoffs, thus evaluating the use of these tools in the screening or diagnosis of all levels of OSA. We also found 2 other systematic reviews with similar aims to ours, but they only featured articles published until 2017. The studies by Mendonça et al [9] and Rosa et al [11] included an extensive array of new digital tools, some still in the research project phase, and others already commercially available. Nevertheless, both reviews included studies without polysomnography as the gold standard, allowing the use of HSAT as a comparison and reference test.

Of the 41 included studies, 7 were smartphone-based tools; 10 were wearables; 11 used bed or mattress sensors; 5 measured nasal airflow; and 8 used other technologies such as radar devices, adhesive patches, or microphones. Out of all of them, only 8 performed external validation of the developed digital tool, whereas 27, the majority, merely performed internal validation. In addition, it is worth mentioning that 8 studies did not present discrimination measures. Regarding internal validation studies, most included bootstrapping or cross-validation techniques.

For the group of studies that only performed internal validation, the one with the highest reported accuracy was that of Norman et al [50]. They used a foam mattress (*Sonomat*) that, aside from recording breath sounds, has pressure sensors that generate voltage when deformed, thus detecting respiratory movements and classifying OSA events. The highest AUC, sensitivity, and specificity values were 1.00, 100%, and 96%, respectively, for a clinical cutoff AHI≥30. When looking at the studies that externally validated the proposed tools, the study that arose as the best, by Wang et al [25], showcased AUC, sensitivity, and specificity values of 0.99, 96%, and 92%, respectively, for an AHI≥30. The proposed tool consists of a noncontact audio recorder that records sleep sounds, which are then analyzed using a deep learning technique that automatically detects sleep apnea events, calculates AHI, and identifies OSA. An overall note should be made for the studies that used mattress sensors, as they revealed some of the best sensitivity, specificity, and AUC values among those that only performed internal validation. In turn, nasal airflow devices showed high specificity, but lacked sensitivity.

On the basis of the currently available published data, contactless devices, such as audio recorders jointly with machine learning techniques, were also shown to have the most significant potential for screening, diagnosis, and possibly monitoring OSA, being a promising path forward. Future work can follow this strategy to further validate these tools because they are still in the development and testing phases.

Clinical questionnaires, such as the STOP-Bang, Berlin, and NoSAS (Neck, Obesity, Snoring, Age, Sex) scores, can help identify patients at increased risk of OSA [3]. Although they are easy to perform and validate in different populations, they do not offer any advantages over digital clinical tools. Given the possibility of day-to-day use and signal recording, the latter can improve the long-term characterization and follow-up of individuals with sleep-breathing disorders. On the basis of the sensitivity and specificity values, STOP-Bang reached 84% and 54%, respectively [62]. For primary care patients, the Berlin questionnaire achieved values of 86% and 77%, respectively [63]. Finally, in the general population, the NoSAS score had sensitivity and specificity values ranging from 79% to 85% and 69% to 77%, respectively [64]. When comparing this with our best results (internally or externally validated), we can see that most digital clinical tools achieved higher sensitivity and specificity values (eg, the best externally validated tool had 96% sensitivity and 92% specificity).

It is important to consider the limitations and strengths of our methodology as well as those of the included studies. Although we cannot be certain that we retrieved all the published literature on the topic, we are confident that our methodology is adequate. The fact that the search was performed in 3 different search engines (one related to health sciences and 2 with a broader spectrum) minimized this risk. Furthermore, it is worth noting that a great part of the available work has substantial gaps in terms of the study design. Although all studies performed an appropriate statistical analysis, many lacked a satisfactory number of participants in both the test and validation groups. At the time of patient enrollment, the reasons for performing polysomnography were also not clear in all manuscripts. The prevalence of OSA varies from 51% to 100%, with some studies not describing this proportion. In addition, most studies evaluated symptomatic patients referred to a sleep clinic and did not reflect the prevalence of OSA in the overall population. Crucial measures to assess diagnostic capabilities, such as sensitivity, specificity, and AUC, have often not been reported. Nearly all digital tools were tested in a controlled laboratory setting, and given the potential use of these devices as an accessible and less expensive method to screen for OSA, it is paramount to invest in further research to test their performance at the home level, where multiple factors might reduce the accuracy of such technologies. Moreover, most studies have developed and tested these devices, but external validation is still lacking. Given the paucity of studies with a comparison with formal in-laboratory polysomnography, additional studies should also be performed in an attempt to validate such digital clinical tools.

Nevertheless, after analyzing the Joanna Briggs Institute checklist results, we believe that we face an overall considerably low risk of bias in both domains. The most common reason for a high risk of bias was the lack of an OSA definition or cutoff for which the discrimination measures were calculated (Q5). However, most studies that did not present the definition did not report the discrimination measures of interest. In addition, a considerable number of studies selected different cutoff values from those described in the guidelines, which makes it difficult to compare the results with similar studies. Another reason was that several designs were faltered in Q3: “Did the study avoid inappropriate exclusions?*”* This is mostly because of an imprecise description of the reasons that led to those exclusions, as in one study where patients with back pain were excluded because this would make the use of the mattress sensor more difficult, which can overestimate the applicability of this type of tool [47]. Moreover, several studies did not include the results for all selected patients (P10), stating that it was attributable to a lack of space [29] or unreturned devices [54]. Even so, it is important to mention that we used a low threshold to consider the answer to the questions as “no” or “unclear.”

### Conclusions

Sleep medicine is a prime field for the use of digital tools and novel unobtrusive technologies. Although they hold great promise, they are still in an early stage of development. This systematic review sheds light on the potential of such devices for the screening or diagnosis of OSA, as they are probably the future of research and development in this field. Although they cannot replace the gold standard of polysomnography, they can greatly assist in large-scale screening and increase the accessibility of the general population to sleep studies. Despite the promising results, this study also highlights the need for future high-quality studies, more robust clinical data, and strategies for care implementation, with the validation of the developed tools in external populations and home environments before they can be used and recommended in a clinical setting.

## Appendix

Multimedia Appendix 1 Search queries used for each database—PubMed, Scopus, and Web of Science.

## Abbreviations

AHI: apnea-hypopnea index
AUC: area under the curve
HR: heart rate
HSAT: home sleep apnea test
NoSAS: Neck, Obesity, Snoring, Age, Sex
OSA: obstructive sleep apnea
PRISMA: Preferred Reporting Items for Systematic Reviews and Meta-Analyses
