# Supplementary material for: The Role of Novel Digital Clinical Tools in the Screening or Diagnosis of Obstructive Sleep Apnea: Systematic Review
Source: J Med Internet Res. 2023 Jul 26;25:e47735. doi: 10.2196/47735 (PMC10413091; doi:10.2196/47735)
Supplement: Multimedia Appendix 1 [file jmir_v25i1e47735_app1.docx]

**Supplementary material**

**Table S1:** Search queries used for each database - PubMed, Scopus, and Web of Science.

| Database | Query |
| --- | --- |
| PubMed | (Apnea, obstructive sleep [MeSH Terms] OR Apnea, obstructive sleep [All Fields] OR obstructive sleep apnea [All Fields] OR OSA [All Fields] OR OSAS [All Fields] OR OSAH [All Fields] OR breathing, sleep disordered [MeSH Terms] OR breathing, sleep disordered [All Fields] OR sleep disorder breathing [All Fields] OR sleep disturbed breathing [All Fields] OR sleep apnea [All Fields]) AND (smartphone [All Fields] OR health applications [All Fields] OR mhealth apps [All Fields] OR mobile health application* [All Fields] OR mobile medical application* [All Fields] OR sleep tracking devices [All Fields] OR sleep trackers [All Fields] OR sleep monitoring device [All Fields] OR wearable device [All Fields] OR wearable sensors [All Fields] OR wearable medical sensors [All Fields] OR mobile healthcare [All Fields] OR wearables [All Fields] OR gadgets [All Fields] OR handheld device [All Fields] OR cellular phone [All Fields] OR smartwatch [All Fields]). |
| Scopus | (TITLE-ABS-KEY (“obstructive sleep apnea”) OR TITLE-ABS-KEY ("sleep apnea, obstructive") OR TITLE-ABS-KEY ("OSA") OR TITLE-ABS-KEY ("OSAS") OR TITLE-ABS-KEY ("OSAH") OR TITLE-ABS-KEY ("breathing, sleep disordered") OR TITLE-ABS-KEY ("sleep disorder breathing") OR TITLE-ABS-KEY ("sleep disturbed breathing") OR TITLE-ABS-KEY ("sleep apnea")) AND (ALL ("smartphone") OR ALL ("health applications") OR ALL ("mhealth apps") OR ALL ("mobile health application*") OR ALL ("mobile medical application") OR ALL ("sleep tracking devices") OR ALL ("sleep trackers") OR ALL ("sleep monitoring device") OR ALL (“wearable device”) OR ALL (“wearable sensors”) OR ALL (“wearable medical sensors”) OR ALL (“mobile healthcare”) OR ALL (“wearables”) OR ALL (“gadgets”) OR ALL (“handheld device”) OR ALL (“cellular phone”) OR ALL (“smartwatch”)). |
| Web of Science | (ALL=(“sleep apnea, obstructive”) OR ALL=(“obstructive sleep apn*”) OR ALL=(“obstructive sleep apnea-hypopnea*”) OR ALL=(“obstructive sleep apnoea-hypopnoea*”) OR ALL=(“apnea-hypon*”) OR ALL=(“OSA”) OR ALL=(“OSAS*”) OR ALL=(“OSAH*”) OR ALL=(“sleep apn*”) OR ALL=(“breathing, sleep disordered”) OR ALL=(“sleep disorder breathing”) OR ALL=(“sleep apnea*”)) AND (ALL=("smartphone") OR ALL=("health applications") OR ALL=("mhealth apps") OR ALL=("mobile health application*") OR ALL=("mobile medical application") OR ALL=("sleep tracking devices") OR ALL=("sleep trackers") OR ALL=("sleep monitoring device") OR ALL=(“wearable device”) OR ALL=(“wearable sensors”) OR ALL=(“wearable medical sensors”) OR ALL=(“mobile healthcare”) OR ALL=(“wearables”) OR ALL=(“gadgets”) OR ALL=(“handheld device”) OR ALL=(“cellular phone”) OR ALL=(“smartwatch”)). |
